# The COVID-19 Infection Did Not Aggravate the Mortality of Long-Term Care Facility Residents Under Strict Infection Control and with Immediate Anti-Viral Treatment: Real-World Analysis

**DOI:** 10.3390/v17050625

**Published:** 2025-04-26

**Authors:** Hideyasu Shimizu, Jin Kawase, Yuko Higashi, Hiroyuki Nabeno, Masamichi Hayashi, Kazuyoshi Imaizumi, Yuji Ito, Masaaki Matsunaga, Mitsushi Okazawa

**Affiliations:** 1 Department of Medicine, Toshiwakai Clinic, Nagoya 460-0022, Japan; hs1029@fujita-hu.ac.jp; 2 Department of Surgery, Toshiwakai Clinic, Nagoya 460-0022, Japan; kawase@toshiwa-kai.or.jp; 3 Department of Nursing, Toshiwakai Clinic, Nagoya 460-0022, Japan; higashi@toshiwa-kai.or.jp (Y.H.); nabeno@toshiwa-kai.or.jp (H.N.); 4 Department of Respiratory Medicine, Fujita Health University School of Medicine, Okazaki Medical Center, Okazaki 444-0827, Japan; michi@fujita-hu.ac.jp; 5 Department of Respiratory Medicine, Fujita Health University School of Medicine, Toyoake 470-1192, Japan; seanluk@fujita-hu.ac.jp; 6 Department of Respiratory Medicine, Daiyukai General Hospital, Daiyukai Health System, Ichinomiya 491-8551, Japan; j48yugmgc@gmail.com; 7 Department of Public Health, Fujita Health University School of Medicine, Toyoake 470-1192, Japan; mm-223@fujita-hu.ac.jp

**Keywords:** COVID-19, SARS-CoV-2, Omicron variant, long-term care facility (LTCF), survival curve

## Abstract

Background: Long-term care facilities (LTCFs) remain highly vulnerable to COVID-19. Despite reduced virulence, Omicron’s high transmissibility poses ongoing risks. The effect of infection under strict control measures and early antiviral treatment remains unclear. Methods: We conducted a retrospective cohort study in a 450-bed LTCF, which implemented rigorous infection control and early antiviral use, evaluating survival outcomes during repeated Omicron outbreaks from January 2022 to December 2023 using Cox regression with time-dependent covariates, adjusted for age, sex, comorbidities, and vaccination status. Mortality trends were also compared across three periods: pre-COVID-19 (2018–2019), COVID-19 present in Japan but absent in our facility (2020–2021), and the Omicron outbreak period (2022–2023). Results: Among 623 residents, 253 were infected. Mortality was lower in the infected group than in the uninfected group (16% vs. 26%), and infection was not significantly associated with increased mortality (HR = 1.36; 95% CI: 0.91–2.04; *p* = 0.14). Although stratified analysis showed higher mortality among infected females, overall mortality during the outbreak period was unexpectedly lower than in prior periods. Conclusions: In LTCFs with rigorous infection control and early antiviral use, Omicron infection did not raise mortality. Enhanced protocols may have improved survival, even among uninfected residents.

## 1. Introduction

The COVID-19 pandemic has resulted in over 704 million infections and 7.0 million deaths globally [1,2]. Since its initial emergence in December 2019, SARS-CoV-2 has undergone numerous mutations, allowing for the rise of several variants of concern (VOCs) [3,4]. The Omicron variant, classified as the fifth VOC in November 2021, quickly became the dominant strain worldwide due to its enhanced transmissibility and ability to evade both natural and vaccine-induced immunity [5,6]. The high number of mutations in the Omicron variant, especially in the spike protein, facilitated its rapid global spread and raised concerns regarding therapeutic resistance [7,8]. Although Omicron has generally been associated with milder clinical outcomes compared to previous variants [9], its explosive surge in cases has continued to strain healthcare systems, economies, and social structures [10].

The emergence of the Omicron variant marked a shift in the pandemic landscape, characterized by a notable increase in infectivity but a significant reduction in mortality [11]. While the number of infections surged during the Omicron wave, both the overall mortality rate and the case fatality rate significantly declined compared to previous waves [12]. Among the populations most severely impacted by COVID-19 are the elderly, particularly those residing in care homes. Life expectancy among care-home residents in Scotland declined notably during the pandemic, reaching its lowest point in 2019/2020 [13]. In the midwest of the United States, the fatality rate for individuals aged 65 and older was 1.03%, leading to a loss of 34 days in life expectancy [14]. A hypothetical scenario of 1 million COVID-19 deaths in the U.S. would lead to a 2.94-year decrease in life expectancy for 2020, with those dying losing an average of 11.7 years of expected remaining life [15].

Recent studies have underscored the critical importance of timely diagnosis and treatment in determining COVID-19 outcomes [16,17]. Delays between symptom onset and diagnosis, as well as between diagnosis and treatment, are significant predictors of disease progression to pneumonia and hospitalization, particularly in high-risk patients [18]. Early diagnosis is associated with improved prognosis, with patients diagnosed within 5 days of symptom onset experiencing better outcomes [19]. Timely intervention is also associated with faster disease resolution and lower CT scores, indicating less severe lung involvement [20]. These findings emphasize the urgent need for easy access to medical care and prompt antiviral treatment to prevent disease progression and hospitalization in high-risk patients [18].

In this observational study, we report on a COVID-19 cluster that occurred in a geriatric care facility. Our investigation evaluated the impact of rapid diagnosis and immediate treatment initiation on short-term COVID-19 outcomes. Additionally, we compared survival rates between infected and uninfected individuals during the same period. Furthermore, we analyzed survival rates across three distinct periods: prior to the COVID-19 pandemic (2018–2019; Period 1), during the pandemic when no cases were reported in the facility (2020–2021; Period 2), and during the Omicron cluster period (2022–2023; Period 3).

## 2. Materials and Methods

This retrospective cohort study was conducted at Toshiwakai, a 450-bed long-term care facility (LTCF) in Nagoya City, Aichi Prefecture, Japan. We analyzed clinical and survival data for all residents between January 2018 and December 2023, covering three distinct periods: (1) pre-COVID-19 era (2018–2019); (2) pandemic era prior to in-facility outbreaks (2020–2021); and (3) the Omicron outbreak period (2022–2023), during which multiple COVID-19 clusters occurred at the facility. Residents who stayed across consecutive periods were counted in each period and included in the analysis for comparative purposes. The study was approved by the Daiyukai Health System Ethics Committee (Approval No. 2022e006) and conducted in accordance with the Declaration of Helsinki.

### 2.1. Prevention and Diagnostic Protocols

With the onset of the COVID-19 pandemic in Japan in 2020 [21], strict preventive measures were implemented in Toshiwakai LTCF. Direct visits by family members were restricted, and all residents and nursing staff were obligated to wear masks and undergo monthly SARS-CoV-2 PCR testing via nasal swabs. Nursing staff were deemed non-infected only after four consecutive antigen tests confirmed negative results for all close-contact colleagues and residents under their care. From the onset of the COVID-19 cluster in 2022, measures to separate symptomatic individuals from asymptomatic individuals were further reinforced. Residents exhibiting symptoms such as fever, upper respiratory tract issues, respiratory symptoms, neurological symptoms, or gastrointestinal symptoms were immediately isolated in private rooms or comparable facilities. Up to four diagnostic tests, including PCR or NEAR, were conducted at approximately 12 h intervals, as clinically indicated. Close-contact residents of suspected cases were subjected to the same diagnostic testing protocols. To minimize transmission risk, infected residents were provided with meals and daily cleaning support separately from uninfected residents, and their movement in the facility was restricted.

### 2.2. Treatment Protocols

Treatment was promptly initiated upon detection of infection, following the protocols outlined below: Remdesivir (200 mg once intravenously on day 1, 100 mg on days 2 and 3), Nirmatrelvir/Ritonavir (300 or 600 mg per day depending on renal function, orally for 5 days), and Molnupiravir (1600 mg per day orally for 5 days). In two cases, Nirmatrelvir/Ritonavir (600 mg per day orally) was supplemented with either Sotrovimab (500 mg IV, single dose) or Casirivimab (600 mg IV, single dose).

### 2.3. Comparison of Demographic Parameters

The degree of care was originally categorized by the Japanese government’s long-term care insurance system [22]. For the purposes of this study, we reclassified it into three categories: low, moderate, and high. Specifically, the “low” category included support levels and level 1; “moderate” included levels 2 and 3; and “high” included levels 4 and 5. The Charlson Comorbidity Index was calculated based on the original scoring system [23]. Additionally, the number of COVID-19 vaccinations received before the start of period 3 was compared between groups.

Voluntary COVID-19 vaccination began in Japan in April 2021. The first and second doses were administered approximately 3 to 4 weeks apart, followed by a booster (third dose) about 6 months later. Subsequent booster doses were administered at intervals of approximately 6 months. Because the number of vaccinations varied among infected residents depending on the timing of infection during period 3, direct comparison with uninfected residents was not feasible. Therefore, the number of vaccinations received before the start of period 3 was compared between the infected and uninfected groups.

### 2.4. Comparison of Symptoms

Symptoms during COVID-19 infection were extracted from daily logs and categorized into six groups: fever, upper respiratory tract symptoms, respiratory symptoms, neurological symptoms, fatigue, and gastrointestinal symptoms. The day treatment commenced (i.e., the day infection was detected) was designated as day 0. Symptom presence was subsequently assessed on days 3, 7, and 10, based on admission logs. Fever was defined as an increase in body temperature of 0.8 °C or more compared to the baseline temperature recorded approximately 2 weeks prior, in the absence of symptoms. For patients with elevated body temperature, the progression of temperature change from day 0 to day 10 was analyzed. Upper respiratory symptoms included a new onset of sore throat, nasal congestion, or hoarseness. Respiratory symptoms included the new onset of coughing, sputum production, or wheezing. Neurological symptoms included a new onset of headache or taste disturbances. Fatigue was recorded if newly developed, and gastrointestinal symptoms included the new onset of diarrhea, vomiting, or loss of appetite. To reduce bias due to acute mortality and care outside the facility, residents who died or were hospitalized within 10 days of infection onset were excluded from the symptom analysis. In deceased cases, the cause of death was often unclear, and non-COVID-related conditions such as advanced comorbidities or frailty may have played a role. In hospitalized cases, although COVID-19 was likely involved, it was not possible to assess the impact of the LTCF’s treatment protocols following transfer. This exclusion allowed for consistent assessment of treatment response over a full 10-day observation period within the LTCF.

### 2.5. Survival Time Comparison

Survival times were compared between infected and uninfected residents within period 3. Additionally, survival times were compared across periods 1 to 3. The residents who admitted to more than two times were counted separately for inter-period comparison. For both the inter-period comparison and the period 3 subgroup comparison, the index date for survival calculation was defined as the start date of the respective study period (i.e., 1 January 2018 for Period 1, 1 January 2020 for Period 2, and 1 January 2022 for Period 3) or the resident’s admission date, whichever occurred later. Observation continued until the date of death. Residents were censored at the date of discharge from the facility or at the end of the respective analysis period, whichever occurred first. Specifically for the Period 3 subgroup comparison, which focused on the first infection episode, residents were also censored at the time of diagnosis of a second COVID-19 infection

### 2.6. Statistical Analysis

Statistical analyses were conducted as follows: Age was expressed as the mean ± standard deviation (SD), with group differences assessed using one-way analysis of variance (ANOVA). The male-to-female ratio across groups was compared using the Chi-square test. Symptom development was summarized as percentages, and comparisons between day 0 and day 10 were performed using McNemar’s test with Holm’s correction for multiple comparisons. Survival rate, vaccination count, and Charlson Comorbidity Index (CCI) were also reported as mean ± SD, and intergroup comparisons were analyzed using one-way ANOVA. The required level of care was presented as a percentage and compared across groups using the Chi-square test. Survival time between infected and uninfected groups in Period 3 was illustrated using Simon and Makuch’s modified Kaplan–Meier curves [24], with comparisons limited to the first infection episode and analyzed using the Mantel–Byar test. Multifactorial analysis of survival time for this comparison was conducted using a time-dependent Cox regression model [25,26], adjusted for age, sex, degree of care, CCI, and number of vaccinations. Survival across periods 1 through 3 was visualized with standard Kaplan–Meier plots and compared using the log-rank test, followed by Cox regression analysis adjusted for age, sex, degree of care, and CCI. Holm’s method was applied to adjust for multiple comparisons between periods. Additionally, in the period3 subgroup comparison, sensitivity analyses were performed by stratifying by age groups (<85 years, 85–90 years, and >90 years), sex. Furthermore, to investigate the relationship between comorbidities and mortality, mean Charlson Comorbidity Index (CCI) scores were compared between residents who survived and those who deceased within each study period. The independent association between CCI score and mortality was assessed using a multivariable Cox regression model, adjusting for age and sex. All statistical analyses were performed using R version 4.2.2 (R Foundation for Statistical Computing, Vienna, Austria). Statistical significance was defined as *p* < 0.05.

## 3. Results

The Japanese government designated COVID-19 as an infectious disease, requiring mandatory daily reporting of new cases and deaths, and all costs were covered by law on 7 February 2020. Up until 8 May 2023, when the reporting system was revised, Japan experienced eight waves of COVID-19. From that date onward, weekly reports of new cases from 70 sentinel clinics in the City of Nagoya were used for surveillance. During period 3, spanning from 1 January 2022, to 31 December 2023, the City of Nagoya experienced significant surges in COVID-19 cases across the sixth-to-ninth infection waves. The cumulative number of deaths reached 417 out of 43,905 new patients by the end of the fifth wave. After the emergence of the sixth wave, characterized by the predominance of the Omicron variant, an additional 1079 fatalities occurred among 638,113 reported cases up until the eighth wave.

**Figure 1:**
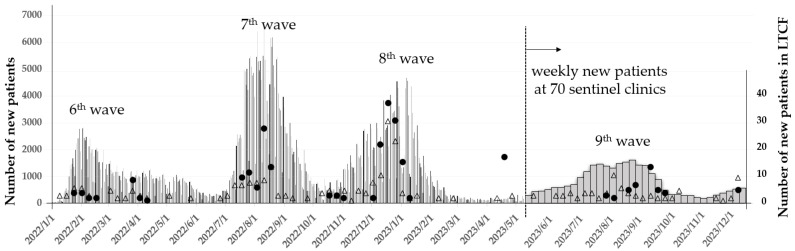
Number of new patients of COVID-19 in Nagoya city and Toshiwakai Long-Term Care Facility. Thin lines show daily new patients. Thick bars show new patients at the 70 sentinel clinics from 9 May 2023, when designation of COVID-19 was ranked down by law, and total number was not counted. Closed circles and open triangles show the weekly new patients of residents and care workers in Toshiwakai Long-Term Care Facility, respectively.

Correspondingly, the Toshiwakai Long-Term Care Facility (LTCF) experienced outbreaks that were initially triggered by care workers (represented by open triangles) and subsequently spread to residents (represented by closed circles) (Figure 1).

A total of 255 care workers and 231 residents were diagnosed with SARS-CoV-2 through antigen testing, PCR, or NEAR testing. Among the infected residents, 210 experienced a single infection episode, 20 had two episodes, and one resident had three episodes, resulting in a cumulative total of 253 confirmed infection episodes.

Of these episodes, 145 (57.3%) involved symptomatic residents, while 108 (42.7%) remained asymptomatic. For the 145 symptomatic episodes, the median interval from symptom onset to diagnosis was 0 min (IQR: 0–55), although six patients required 24–46 h for infection confirmation. Treatment was initiated immediately upon diagnosis, including during night shifts. Antiviral monotherapy was administered as follows: Remdesivir in 145 episodes, Nirmatrelvir/Ritonavir in 78 episodes, and Molnupiravir in 30 episodes. Combination therapy was employed in three episodes: Nirmatrelvir/Ritonavir with Sotrovimab or Casirivimab/Imdevimab in two episodes, and Remdesivir with Casirivimab/Imdevimab in one episode.

Among the infected residents, four patients aged 88–93 years died within 10 days, two of whom were in terminal stages prior to infection onset, and one was diagnosed 46 h from the onset of the fever. Additionally, five patients required hospital admission within 10 days of infection: two for fall-related fractures and three for dehydration. Excluding these nine patients, a total of 244 infection episodes were analyzed over a 10-day period.

### 3.1. Symptom Profile and Acute Outcomes

**Figure 2:**
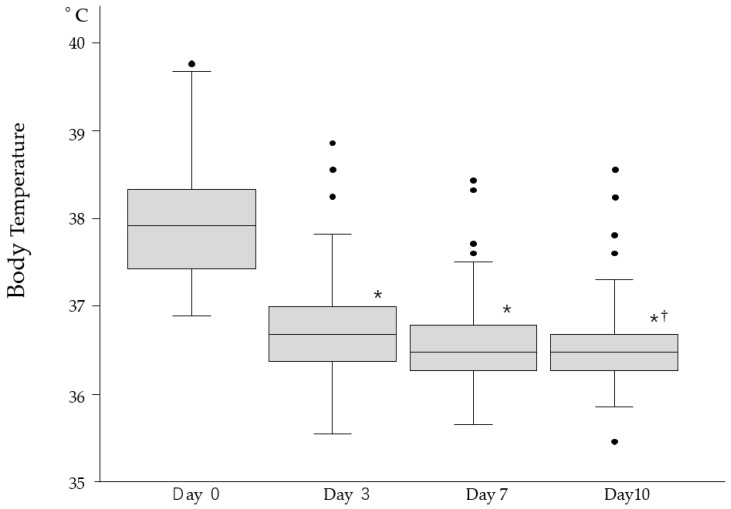
Comparison of body temperature (BT) in febrile patients (n = 109) following antiviral treatment. BT significantly decreased by day 3 compared to day 0 (* *p* < 0.001) and further decreased by day 10 compared to day 3 (^†^ *p* < 0.05). Black dots represent individual outliers beyond the interquartile range.

Fever was the most common (44.3%), followed by respiratory symptoms (16.8%) and upper airway symptoms (10.2%) (Table 1). Symptoms resolved in most residents within 10 days, although 13.9% experienced prolonged or residual symptoms such as cough, fatigue, or low-grade fever. Figure 2 illustrates fever patterns across all infected episodes. Most fevers subsided within 3 days, and body temperatures normalized by day 10 in nearly all cases. Four residents died within 10 days of a COVID-19 diagnosis, and three required hospitalizations. These acute-phase outcomes were analyzed separately from long-term survival analyses to avoid attribution bias.

### 3.2. Survival Analysis: Infected vs. Uninfected

During period 3, when the Toshiwakai LTCF experienced a surge in COVID-19 cases (Figure 1), residents were divided into infected (n = 231) and uninfected (n = 392) groups for comparison. The characteristics of these two groups are summarized in Table 2.

The total number of all-cause deaths was significantly lower in the infected group (16%) compared to the uninfected group (26%, *p* = 0.004). Although age, care burden, and Charlson Comorbidity Index (CCI) were comparable between groups, the proportion of males was significantly lower in the infected group (*p* < 0.001). The number of vaccinations differed significantly between groups; 77% of infected residents received two vaccinations, compared to 61% in the uninfected group (*p* < 0.001).

**Figure 3:**
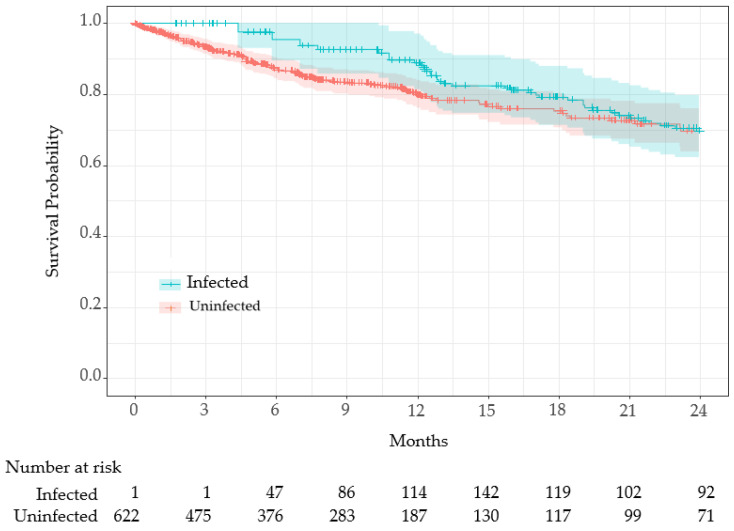
Simon and Makuch’s Kaplan–Meier curves comparing survival rates between infected and uninfected residents during period 3. The shaded areas (blue and red) represent the 95% confidence intervals for each group. Time-dependent Cox proportional hazards analysis demonstrated no statistically significant difference in survival between the two groups.

COVID-19 infection was treated as a time-dependent variable, and survival time was compared using Simon and Makuch’s modified Kaplan–Meier curves (Figure 3).

Since all residents (n = 623) were uninfected at the beginning of period 3 (1 January 2022) or at the time of admission, the entire cohort was initially at risk. As uninfected residents transitioned to the infected group, the number of at-risk infected residents increased. The Mantel–Byar test indicated no significant difference in survival between groups (*p* = 0.42).

The time-dependent Cox proportional hazards analysis for period 3 is shown in Table 3. In the univariate analysis, COVID-19 infection was associated with a hazard ratio (HR) of 1.43, but this was not statistically significant (*p* = 0.08). In the multivariate analysis, which accounted for age, sex, care level, CCI, and number of vaccinations, COVID-19 infection had an HR of 1.36, though it remained non-significant (*p* = 0.14). Age and sex were significant risk factors, with age showing an HR of 1.07 (*p* < 0.001) and male sex showing an HR of 3.25 (*p* < 0.001). Care level “high” was also a significant risk factor, with an HR of 3.66 (*p* = 0.005). While the number of vaccinations reduced the HR by 17%, this effect was not statistically significant (*p* = 0.09).

#### 3.2.1. Stratified Survival Analysis by Age

Survival analyses were conducted separately for three age groups: <85 years, 85–90 years, and >90 years (Supplemental Tables S4, S8 and S12). In all three strata, mortality was lower in period 3 (Omicron era) compared to period 2 (pre-outbreak period). The difference was statistically significant in the 85–90 age group (HR = 0.49; 95% CI: 0.30–0.80; *p* = 0.008). The <85 and >90 age groups also showed lower hazard ratios in period 3 compared to period 2, but these did not reach statistical significance.

#### 3.2.2. Stratified Analysis by Sex

Sex-stratified survival analysis revealed differing trends. Infected females had a significantly increased risk of mortality compared to uninfected females (HR = 1.62; 95% CI: 1.03–2.54, *p* = 0.035; Supplemental Table S18), while infected males had a nonsignificant trend toward reduced mortality (HR = 0.50; 95% CI: 0.16–1.55, *p* = 0.23; Supplemental Table S14).

#### 3.2.3. Stratified Survival Analysis by Survival Status and CCI

To investigate the relationship between comorbidities and mortality, we stratified residents into survived and deceased groups and analyzed their Charlson Comorbidity Index (CCI) across the three study periods (Supplemental Table S19). In all periods, deceased residents had higher mean CCI scores than survivors. This difference was most pronounced in period 3, where the mean CCI was 1.58 in deceased residents versus 1.30 in survivors. Multivariable Cox regression confirmed that higher CCI scores were independently associated with increased mortality in period 3 (HR = 1.16; 95% CI: 1.00–1.34; *p* = 0.045; Supplemental Table S22), even after adjusting for age, sex. However, this association was not statistically significant in periods 1 or 2 (Supplemental Tables S20 and S21).

### 3.3. Comparison Between Periods

For the inter-period analysis, each resident admission was treated as a separate observation if the individual was admitted more than once during different periods. As a result, the total number of residents in the period comparison (e.g., 630 in period 3 for Table 4) slightly exceeds the unique resident count used in Table 2 and Figure 3 (n = 623).

**Figure 4:**
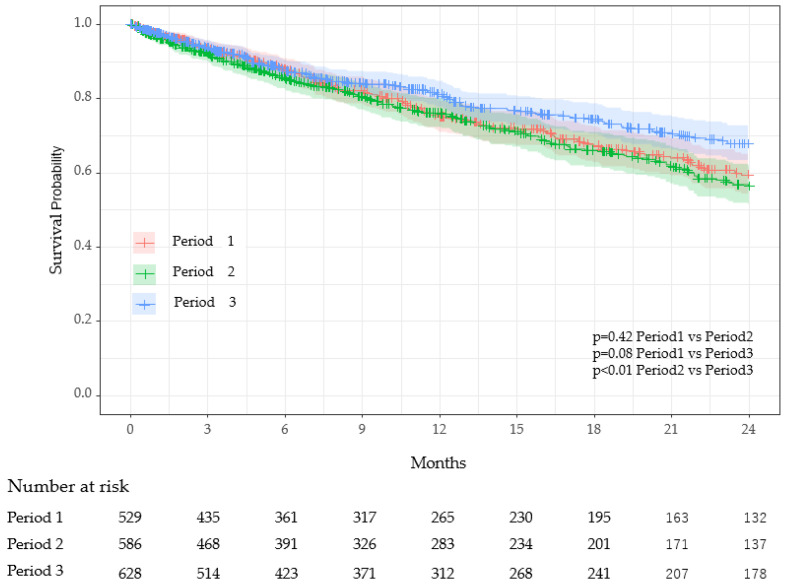
The Kaplan–Meier survival curves with 95% confidence intervals across three distinct periods. Cox proportional hazards analysis indicated no significant differences in survival between period 1 vs. period 2 and period 1 vs. period 3. However, a statistically significant difference was observed between period 2 and period 3 (*p* < 0.01). *p*-value was obtained by multiple comparisons using the Holm test.

Table 4 presents an inter-period comparison of key characteristics. COVID-19 infections among residents occurred exclusively in Period 3, during which 210 residents experienced a single episode, 20 had two episodes, and one had three episodes. There were no significant differences in age, sex, or person–years between periods. However, the number of deaths was significantly lower in period 3 (22%) compared to period 1 (28%) and period 2 (29%), with adjusted *p*-values of 0.012 (period 1 vs. period 3) and 0.045 (period 2 vs. period 3). The CCI was significantly higher in period 1 (mean 1.60) compared to period 2 (1.37) and period 3 (1.36), with adjusted *p*-values of 0.005 (period 1 vs. period 2) and 0.004 (period 1 vs. period 3). Survival curves using Kaplan–Meier plots revealed significant differences in survival probabilities between period 2 and period 3 (adjusted *p* < 0.01), while no significant differences were observed between period 1 and period 2 or period 1 and period 3 (Figure 4).

Cox proportional hazards analysis showed that, compared to period 2, the hazard in period 3 was significantly reduced by 27% (*p* = 0.02) (Table 5). An increase in care level significantly elevated the hazard; care level “moderate” increased the hazard by 56% (*p* = 0.035), and care level “high” increased it by 199% (*p* < 0.001). Although CCI tended to increase the hazard, this trend was not statistically significant.

## 4. Discussion

Long-term care facilities (LTCFs) are particularly vulnerable to COVID-19 cluster outbreaks. Before the Omicron surge in 2021, a meta-analysis reported that the incidence rate among LTCF residents was 45% per facility, with a mortality rate of 23% [27]. The emergence of the Omicron variant and its sublineages dramatically altered the infection landscape. While infectivity significantly increased, the severity and mortality rates declined [28,29], resulting in explosive transmission, particularly within closed environments such as LTCFs. Although the individual case-fatality rate dropped, the total number of deaths among elderly residents rose significantly. A press release from the Nagoya city government reported that the cumulative number of deaths prior to the emergence of Omicron (up to December 2021) was 471, with a mortality of 1.04%. However, during the subsequent 1.5 years, when Omicron was dominant, the number of deaths rose to 1079 despite the mortality rate decreasing to 0.17%. Notably, over 90% of these fatalities were among individuals aged 60 years or older. The first case of COVID-19 at the Toshiwakai LTCF was a care worker, identified on 24 December 2020. Four additional care workers tested positive in 2021 before the Omicron variant emerged, but no cases were detected among residents. However, after a care worker was diagnosed with COVID-19 on 15 January 2022, likely due to Omicron, the number of resident infections surged dramatically starting 4 February 2022 (Figure 1). Because in-person visits by family had already been restricted since 2020, it is likely that the virus was introduced by care workers. During the seventh and eighth waves, the weekly number of new cases of residents and care workers reached 100, leading to severe disruptions in LTCF operations. Serial antigen testing, previously reported as effective for detecting new cases [30], was implemented repeatedly. This allowed us to identify 108 asymptomatic cases among 253 total infection episodes (42.7%). The increased urgency during this period heightened the vigilance of care workers and led to stricter isolation protocols for symptomatic patients. Since early outpatient treatment has been shown to mitigate disease progression [18], antiviral therapy was initiated immediately upon confirmation of SARS-CoV-2 infection, even during night shifts. Due to Omicron’s shorter incubation and serial intervals compared to previous variants [31,32], early detection and rapid intervention were considered essential to suppress transmission and prevent severe illness.

In our study, 59% of infected residents developed symptoms, with fever being the most prominent, followed by respiratory and upper airway symptoms (Table 1). Most symptoms resolved within 10 days, although 13.9% of residents reported persistent symptoms, primarily respiratory complaints, fever, and fatigue. Figure 2 shows the fever pattern across 108 episodes; while individual variation was observed, fever typically resolved within 3 days and normalized by day 10. During the acute phase of infection (first 10 days), four residents died, and three required hospitalization, likely due to COVID-19. These observations initially led us to hypothesize that COVID-19 infection would increase mortality among LTCF residents. However, crude mortality was significantly lower in the infected group (16%) compared to the uninfected group (26%) during period 3 (Table 2).

During the infection cluster in period 3, stringent infection control protocols were implemented, and antiviral treatments were administered promptly. The infected group also had a significantly higher proportion of female residents compared to males (Table 2). Although the underlying reason for this gender disparity is unclear, it is possible that closer social interactions among women in LTCFs may have facilitated greater transmission compared to men. It is well-documented that women generally have lower mortality risks than men in the general population [33,34,35], and recent findings from the Delta and Omicron eras suggest that women experienced less severe illness compared to men [36,37]. However, our stratified analysis revealed a significantly higher hazard ratio among infected females (HR = 1.62; Supplemental Table S18), while infected males showed a non-significant trend toward lower mortality (HR = 0.50; Supplemental Table S14). This finding may seem counterintuitive, as women are generally known to have a survival advantage over men in both the general population and among COVID-19 patients. One possible explanation is that this survival advantage, although attenuated by frailty or other unmeasured vulnerabilities, still conferred a protective effect on overall mortality. As a result, the higher proportion of females in the infected group may have contributed to its unexpectedly lower crude mortality. These opposing trends between sexes likely offset each other in the multivariable model, leading to a nonsignificant difference in overall survival between infected and uninfected groups.

Although the Omicron variant of SARS-CoV-2 is known to evade neutralizing antibodies induced by current mRNA vaccines [38], vaccination may still have played a role in mitigating infection. In this study, infected residents had received significantly more vaccine doses before period 3 compared to uninfected residents (Table 2). However, this difference may reflect disparities in vaccination opportunities rather than vaccine inefficacy. Because period 3 included both long-term residents and those newly admitted between 1 January 2022, and 31 December 2023, vaccine counts were standardized using a 1 January 2022, cutoff to reduce time-dependent bias. Booster (third) doses were limited due to rollout timing, but the proportion of residents receiving at least two doses was notably higher in the infected group. Despite this unexpected distribution, Cox proportional hazards modeling indicated a trend toward reduced mortality with increasing vaccination (HR = 0.83; *p* = 0.09, Table 3). These findings support the hypothesis that current vaccines, while less effective at preventing Omicron infection, continue to reduce disease severity and mortality [39]. As vaccination efforts continued throughout the 2-year period of period 3, assessing the protective effect of vaccination is challenging, in contrast to previous findings in LTCF populations and immunocompromised individuals [40,41].

To address time-dependent bias caused by heterogeneous infection timing, we used infection status as a time-dependent covariate in the survival analysis [25,26]. Among the factors examined, age, sex, care level, and the Charlson Comorbidity Index (CCI) were identified as significant contributors to mortality, while vaccination demonstrated a protective association, albeit without statistical significance (Table 3). After adjusting for confounding variables, COVID-19 infection was associated with a hazard ratio of 1.36 (95% CI: 0.91–2.04, *p* = 0.14, Table 3), indicating a potential, though statistically non-significant, increase in mortality risk. The survival curve analysis (Figure 3) and Mantel–Byar test similarly showed no significant difference in survival between infected and uninfected groups (*p* = 0.42). These findings suggest that robust infection control and timely therapeutic strategies may effectively offset the survival impact of COVID-19 infection in LTCFs, highlighting the need for further investigation to validate these observations.

Analysis of survival rates across the three study periods yielded unexpected findings. Period 3, which included COVID-19 clusters within the facility, demonstrated significantly better survival compared to period 2 (when no in-facility cases occurred despite national surges), and slightly higher survival than period 1 (before COVID-19 emerged in Japan), though the latter was not statistically significant (Figure 4, Table 5). Stratified age analysis revealed consistently lower hazard ratios in period 3 compared to period 2 across all age groups, with statistical significance reached in the 85–90-year subgroup (Supplemental Tables S4, S8 and S12), suggesting that strengthened infection control and care practices during the Omicron era may have contributed to improved survival. These results contrast with findings by Jones and Ponomarenko, who observed excess mortality during the Omicron period using a single-year age model [42]. Although the hazard ratio increased by 9% from period 1 to period 2 (Table 5), this may be explained by undiagnosed COVID-19 cases due to limited routine testing and standard infection control at the time. Importantly, the Charlson Comorbidity Index (CCI), a well-established predictor of mortality in LTCFs [43], was significantly lower in period 3 residents than in those from periods 1 and 2 (Table 4), potentially contributing to improved survival outcomes. However, this alone is insufficient to account for the magnitude of observed mortality reduction, as deceased residents still exhibited significantly higher CCI scores than survivors (HR = 1.16, 95% CI: 1.00–1.34, *p* = 0.045; Supplemental Table S22). These findings suggest that although the overall CCI was lower in period 3, individual comorbidity burden remained a significant predictor of mortality, particularly during the Omicron era.

Further analysis comparing survival between periods 1 and 3, excluding post-infection follow-up in period 3, revealed a significant survival advantage in period 3 (Supplemental Tables S23 and S24). This finding supports the hypothesis that strengthened infection control protocols during COVID-19 clusters may have helped prevent outbreaks of other respiratory pathogens such as influenza, respiratory syncytial virus (RSV), and human metapneumovirus [44]. Several studies have reported a dramatic reduction in these infections during the pandemic, largely attributed to universal mask use, physical distancing, and enhanced hygiene practices [45,46]. Conversely, their resurgence following the relaxation of these measures underscores the importance of rigorous infection control in mitigating respiratory viral transmission [47,48,49]. These observations emphasize the critical role of integrated infection control policies in improving survival outcomes and safeguarding vulnerable populations during pandemics.

## 5. Conclusions

This study highlights the critical role of comprehensive infection control measures and prompt antiviral therapy in managing COVID-19 outbreaks in long-term care facilities (LTCFs). Despite the highly transmissible Omicron variant, our findings showed no significant increase in mortality among infected residents compared to uninfected residents. The survival rates during the Omicron cluster (period 3) were unexpectedly higher than in previous periods, underscoring the efficacy of interventions such as rigorous infection control protocols. Additionally, our results suggest that enhanced infection control measures not only mitigate COVID-19 outcomes but may also suppress the transmission of other respiratory pathogens. These outcomes reinforce the importance of maintaining robust preventive and therapeutic strategies in high-risk populations, particularly LTCF residents.

## 6. Limitation

This study has several limitations. First, as a single-facility observational study, generalizability may be limited. Second, despite the use of time-dependent covariate modeling, residual confounding due to unmeasured variables, such as individual functional status or cognitive impairment, may persist. Third, vaccine counts were assessed only up to the start of period 3, and changes during the 2-year period were not incorporated into the analysis. Additionally, the exclusion of hospitalized and deceased residents from the symptom analysis may have led to an underestimation of disease severity. Finally, due to the evolving nature of COVID-19 variants and treatment protocols, the observed associations may not be directly extrapolated to future outbreaks.

## Figures and Tables

**Table 1:** Clinical Symptoms following COVID-19 treatment.

Symptoms	Day 0 (n = 244)	Day 3 (n = 244)	Day 7 (n = 244)	Day 10 (n = 244)
High body temperature (%)	108 (44.3)	41 (16.8) *	24 (9.8) *	14 (5.7) *
Upper airway symptoms (%)	25 (10.2)	22 (9.0) *	13 (5.3) *	1 (0.4) *
Respiratory symptoms (%)	41 (16.8)	56 (23.0)	34 (13.9)	19 (7.8) ^†^
Neurological symptoms (%)	10 (4.1)	2 (0.8)	2 (0.8)	0 (0.0) *
General fatigue (%)	1 (0.4)	4 (1.6)	5 (2.1)	6 (2.5)
Abdominal symptoms (%)	3 (1.2)	3 (1.2)	4 (1.6)	3 (1.2)
Any symptoms of above (%)	141 (59.0)	99(41.0)	65 (26.6)	34 (13.9) *

Numbers (percentage) of six symptoms from day 0 (initiation of treatment) to day 10; nine patients out of 253 were excluded for comparison due to their discharge from the facilities within 10 days. McNemar test with Holm’s correction for multiple comparisons, * *p* < 0.0001, ^†^ *p* < 0.01.

**Table 2:** Clinical and Demographic Characteristics by COVID-19 infection status in Period 3.

Characteristics	Uninfected (n = 392) ^1^	Infected (n = 231) ^1^	*p*-Value ^2^
Number of Deaths	102 (26%)	37 (16%)	0.004
Age (years)	89 (7)	88 (6)	0.45
Sex (% of males)	81 (21%)	19 (8.2%)	<0.001
Degree of care			0.78
Low	37 (9.4%)	25 (11%)	
Moderate	187 (48%)	112 (48%)	
High	168 (43%)	94 (41%)	
Charlson comorbidity index	1.24 (0.96)	1.27 (0.94)	0.77
Number of vaccinations			<0.001
0	145 (37%)	50 (22%)	
1	6 (1.5%)	1 (0.4%)	
2	240 (61%)	177 (77%)	
3	1 (0.3%)	3 (1.3%)	

^1^ n (%); Mean (SD), *^2^* Fisher’s exact test; one-way analysis of means (not assuming equal variances).

**Table 3:** Time-dependent Cox regression analysis of COVID-19 infection on Mortality during Period 3.

	Univariate			Multivariate		
Term	Hazard Ratio	95% CI	*p*-Value	Hazard Ratio	95% CI	*p*-Value
COVID-19 infection	1.43	0.95–2.15	0.08	1.36	0.91–2.04	0.14
Age				1.07	1.04–1.11	<0.001
Sex						
Female				1.00	(Ref)	
Male				3.25	2.19–4.83	<0.001
Degree of Care						
Low				1.00	(Ref)	
Moderate				1.82	0.73–4.56	0.20
High				3.66	1.50–8.95	0.005
Charlson comorbidity index				1.10	0.93–1.29	0.26
Number of Vaccinations				0.83	0.68–1.03	0.09

**Table 4:** Comparison of Clinical and Demographic Characteristics across Study Periods.

Characteristics	Period 1 (n = 529) ^1^	Period 2 (n = 589) ^1^	Period 3 (n = 630) ^1^
Number of Infections			
0	529	589	399
1	0	0	210
2	0	0	20
3	0	0	1
Age (years)	88 (7)	89 (6)	89 (6)
Sex (% of male)	85 (16%)	92 (16%)	103 (16%)
Person–year	1.07 (0.73)	1.01 (0.74)	1.07 (0.74)
Number of Deaths	149 (28%)	172 (29%)	139 (22%) *
Degree of care			
Low	63 (12%)	78 (13%)	63 (10%)
Moderate	236 (45%)	270 (46%)	302 (48%)
High	230 (43%)	241 (41%)	265 (42%)
Charlson comorbidity index	1.60 (1.19)	1.37 (1.04) ^†^	1.36 (1.05) ^†§^

^1^ Mean (SD), n (%); * *p* < 0.05 period 3 vs. period 1 and period 2, ^†^ *p* < 0.01 period 1 vs. period 2 and period 3, ^§^ *p* < 0.05 period 2 vs. period 3.

**Table 5:** Cox proportional hazard model for inter-period comparison.

	Univariate			Multivariate		
Term	Hazard Ratio	95% CI.	*p*-Value ^1^	Hazard Ratio	95% C.I.	*p*-Value ^1^
Period 1 vs. Period 2	1.09	0.88–1.36	0.42	1.09	0.88–1.36	0.42
Period 1 vs. Period 3	0.78	0.62–0.99	0.09	0.80	0.63–1.01	0.14
Period 2 vs. Period 3	0.72	0.57–0.90	0.01	0.73	0.58–0.92	0.02
Age				1.05	1.04–1.07	<0.001
Sex				2.47	1.94–3.16	<0.001
Female				1.00	(Ref)	
Male				2.47	1.94–3.16	<0.001
Degree of Care						
Low				1.00	(Ref)	
Moderate				1.56	1.03–2.34	0.035
High				2.99	2.01–4.45	<0.001
CCI				1.07	1.00–1.54	0.065

CCI; Charlson comorbidity Index. ^1^ Holm- adjusted *p*-value.

## Data Availability

All data for statistical analysis were collected from the daily records of Toshiwakai LTCF. The dataset generated and analyzed during this study is available from the corresponding author upon reasonable request.

## Supplementary Materials

The following supporting information can be downloaded at: https://www.mdpi.com/article/10.3390/v17050625/s1.

## Abbreviations

The following abbreviations are used in this manuscript:

LTCF	Long-term care facility
SARS-CoV-2	Severe acute respiratory syndrome coronavirus 2
COVID-19	Coronavirus disease-2019
VOC	Variant of concern
C.I.	Confidence Interval
PCR	Polymerase chain reaction
NEAR	Nicking enzyme amplification reaction
