# Supplementary material for: The COVID-19 Infection Did Not Aggravate the Mortality of Long-Term Care Facility Residents Under Strict Infection Control and with Immediate Anti-Viral Treatment: Real-World Analysis
Source: Viruses. 2025 Apr 26;17(5):625. doi: 10.3390/v17050625 (PMC12115418; doi:10.3390/v17050625)
Supplement: Supplementary file 1 [file viruses-17-00625-s001.zip › viruses-3512196-supplementary.pdf]

Age stratification

Age <85

Table S1 Characteristics for Uninfected and Infected groups in Period 3

Characteristic	Uninfected (n=90)[1]	Infected(n=45)[1]	p-value[2]
Number of deaths	16 (18%)	5 (11%)	0.45
Age	79.4(4.8)	79.7(4.0)	0.76
Sex (% of Male)	29 (32%)	3 (6.7%)	0.001
Degree of care			0.40
low	7 (7.8%)	4 (8.9%)	
Moderate	38 (42%)	24 (53%)	
High	45 (50%)	17 (38%)	
CCI	1.24(0.93)	1.49(1.10)	0.20
Number of vaccination			0.36
0	42 (47%)	18 (40%)	
2	47 (52%)	25 (56%)	
3	1 (1.1%)	2 (4.4%)	

[1] n (%); Mean (SD)

[2] Fisher's exact test; One-way ANOVA (not assuming equal variances)

Table S2 Cox hazard ratio for Period 3 of age<85

	Univariate			Multivariate		
Term	Hazard ratio	95% C.I.	p-value	Hazard ratio	95% C.I.	p-value
COVID-19 infection	1.56	0.65-3.76	0.32	1.58	0.60-4.17	0.36
Age				0.99	0.88-1.12	0.93
Sex				5.82	2.42-13.90	<0.001
Degree of Care						
High				2.77	1.09-7.05	0.031
CCI				0.98	0.74-1.30	0.90
Number of Vaccinations				0.65	0.42-1.02	0.059

Degree of care categories of Low and Moderate was combined and compared with category High

Inter-period comparison of age <85

Table S3 Characteristics of Period 1-3

Characteristic	Period 1 (n=126[1])	Period 2 (n=134) [1]	Period 3 (n=137) [1]	p value[2]
Age	79.7(4.8)	80.3(4.2)	79.6(4.6)	0.33
Sex	32 (25%)	32 (24%)	32 (23%)	0.94
Person-year	1.09(0.77)	0.94(0.73)	0.94(0.74)	0.20
Number of Deaths	16 (13%)	25 (19%)	21 (15%)	0.43
Degree of care				0.12
Low	12 (9.5%)	24 (18%)	12 (8.8%)	
Moderate	54 (43%)	60 (45%)	62 (45%)	
High	60 (48%)	50 (37%)	63 (46%)	
CCI	1.71(1.19)	1.47(1.07)	1.38(1.04)	

Period 1:2018-2019, Period 2:2020-2021, Period 3:2022-2023

[1] Mean (SD); n (%)

[2] One-way analyses of means (not assuming equal variances); Fisher's exact test with Holm test

Table S4 Cox Hazard test for inter-period comparison of age<85

	Univariate			Multivariate		
Term	Hazard ratio	95% C.I.	p-value	Hazard ratio	95% C.I.	p-value
Period 1 vs Period 2	1.62	0.85-3.08	0.42	1.73	0.91-3.29	0.29
Period 1 vs Period 3	1.34	0.69-2.59	0.77	1.41	0.74-2.70	0.59
Period 2 vs Period 3	0.83	0.47-1.46	0.77	0.82	0.45-1.48	0.59
Age				1.01	0.96-5.68	0.72
Sex				3.37	1.99-5.68	<0.001
Degree of Care						
Moderate				4.17	0.55-31.90	0.17
High				11.36	1.50-85.89	0.019
CCI				1.16	0.94-1.43	0.16

Age85-90

Table S5 Characteristics for Uninfected and Infected groups in Period 3

**Characteristic**	Uninfected (n=119)[1]	Infected (n=86)[1]	p-value[2]
Number of deaths	17 (14%)	12 (14%)	>0.99
Age	87.16(1.40)	87.02(1.37)	0.47
Sex (% of Male)	25 (21%)	11 (13%)	0.14
Degree of care			0.015
low	14 (12%)	14 (16%)	
Moderate	63 (53%)	28 (33%)	
High	42 (35%)	44 (51%)	
CCI	1.28(0.95)	1.22(0.87)	0.66
Number of vaccinations			<0.01
0	49 (41%)	17 (20%)	
1	3 (2.5%)	1 (1.2%)	
2	67 (56%)	67 (78%)	
3	0 (0%)	1 (1.2%)	

[1] n (%); Mean (SD)

[2] Fisher's exact test; One-way analysis of means (not assuming equal variances)

Tale S6 Cox hazard ratio for Period 3 of age 85-90

	Univariate 183			Multivariate		
Term	Hazard ratio	95% C.I.	p-value	Hazard ratio	95% C.I.	p-value
COVID-19 infection	2.35	1.02-5.45	0.045	2.21	0.94-5.15	0.067
Age				0.91	0.72-1.15	0.44
Sex				1.76	0.67-4.60	0.25
Degree of Care						
High				2.60	1.18-5.69	0.017
CCI				0.97	0.65-1.46	0.88
Number of Vaccinations				1.06	0.63-1.77	0.84

Degree of care categories of Low and Moderate were combined and compared with category High

Inter period comparison of age 85-90

Table S7 Characteristics of inter-period comparison

Characteristic	Period 1 (n=169[1])	Period 2 (n=180)[1]	Period 3 (n=207)[1]	p value[2]
Age	87.17(1.39)	87.24(1.37)	87.11(1.38)	0.61
Person-year	1.06(0.72)	1.01(0.72)	1.08(0.72)	0.65
Number of Deaths	46 (27%)	43 (24%)	29 (14%)	0.004
Degree of care				0.64
Low	30 (18%)	26 (14%)	28 (14%)	
Moderate	77 (46%)	89 (49%)	93 (45%)	
High	62 (37%)	65 (36%)	86 (42%)	
CCI	1.51(1.08)	1.36(1.05)	1.35(1.03)	0.26

Period 1:2018-2019, Period 2:2020-2021, Period 3:2022-2023

[1] Mean (SD); n (%)

[2] One-way analysis of means (not assuming equal variances); Fisher's exact test

Table S8 Cox Hazard test for inter-period comparison of age 85-90

	Univariate			Multivariate		
Term	Hazard ratio	95% C.I.	p-value	Hazard ratio	95% C.I.	p-value
Period1 vs Period2	0.92	0.60-1.39	0.69	0.90	0.59-1.37	0.62
Period1 vs Period3	0.50	0.32-0.80	0.012	0.44	0.28-0.70	0.002
Period2- vs Period3	0.55	0.34-0.90	0.032	0.49	0.30-0.80	0.008
Age				1.02	0.90-1.16	0.76
Sex				2.26	1.42-3.60	<0.001
Degree of Care						
Moderate				1.62	0.78-3.43	0.20
High				3.74	1.85-7.53	<0.001
CCI				1.06	0.90-1.25	0.48

Age>90

Table S9 Characteristics of Period 3

**Characteristic**	Uninfected (n=183)[1]	Infected(n=100)[1]	p-value[2]
Number of deaths	69 (38%)	20 (20%)	0.002
Age	94.18(3.25)	93.23(2.80)	0.011
Sex (% of Male)	27 (15%)	5 (5.0%)	0.017
Degree of care			0.12
low	16 (8.7%)	7 (7.0%)	
Moderate	86 (47%)	60 (60%)	
High	81 (44%)	33 (33%)	
CCI	1.22(0.98)	1.21(0.92)	0.91
Number of vaccinations			0.006
0	54 (30%)	15 (15%)	
1	3 (1.6%)	0 (0%)	
2	126 (69%)	85 (85%)	

[1] n (%); Mean (SD)

[2] Fisher's exact test; One-way analysis of means (not assuming equal variances)

Table S10 Cox hazard ratio for Period 3 of age >90

	Univariate			Multivariate		
Term	Hazard ratio	95% C.I.	p-value	Hazard ratio	95% C.I.	p-value
COVID-19 infection	0.90	0.53-1.53	0.69	1.13	0.65-1.96	0.67
Age				1.07	0.99-1.14	0.077
Sex				3.66	2.17-6.18	<0.001
Degree of Care						
Moderate				0.80	0.31-2.04	0.64
High				1.64	0.66-4.04	0.28
CCI				1.19	0.97-1.47	0.089
Number of Vaccinations				0.88	0.67-1.17	0.39

Inter-period comparison of age>90

Table S11 Characteristics of inter-period comparison

Characteristic	Period 1 (n=234) [1]	Period 2 (n=275) [1]	Period 3 (n=286) [1]	p value[2]
Age	93.8(3.3)	93.8(3.3)	93.8(3.1)	>0.99
Sex	28 (12%)	30 (11%)	33 (12%)	0.93
Person-year	1.07(0.73)	1.05(0.74)	1.12(0.75)	0.47
Number of Deaths	87 (37%)	104 (38%)	89 (31%)	0.19
Degree of care				0.43
Low	21 (9.0%)	28 (10%)	23 (8.0%)	
Moderate	105 (45%)	121 (44%)	147 (51%)	
High	108 (46%)	126 (46%)	116 (41%)	
CCI	1.59(1.27)	1.32(1.02)	1.36(1.07)	0.027

Period 1:2018-2019, Period 2:2020-2021, Period 3:2022-2023

[1] mean (SD); n (%)

[2] One-way analysis of means (not assuming equal variances); Fisher's exact test

Table S12 Cox Hazard test for inter-period comparison of age>90

	Univariate			Multivariate		
Term	Hazard ratio	95% C.I.	p-value	Hazard ratio	95% C.I.	p-value
Period1 vs Period2	1.04	0.78-1.38	0.80	1.09	0.83-1.44	>0.99
Period1 vs Period3	0.80	0.59-1.08	0.29	0.90	0.67-1.22	>0.99
Period2- vs Period3	0.77	0.58-1.03	0.23	0.82	0.62-1.10	0.56
Age				1.04	1.01-1.08	0.010
Sex				2.35	1.65-3.36	＜0.001
Degree of Care						
Moderate				1.22	0.71-2.08	0.47
High				2.07	1.23-3.49	0.006
CCI				1.07	0.98-1.18	0.15

Gender stratification

Table S13 Characteristics of Male in Period 3

Characteristic	Uninfected (n=81)[1]	Infected (n=19)[1]	p-value[2]
Number of deaths	32 (40%)	3 (16%)	0.063
Age	87(7)	88(6)	0.40
Degree of care			0.30
Low	13 (16%)	2 (11%)	
Moderate	30 (37%)	11 (58%)	
High	38 (47%)	6 (32%)	
CCI	1.58(1.16)	1.63(1.10)	0.90
Number of vaccinations			0.40
0	42 (52%)	7 (37%)	
1	1 (1.2%)	0 (0%)	
2	38 (47%)	12 (63%)	

[1] n (%); Mean (SD)

[2] Fisher's exact test; One-way analysis of means (not assuming equal variances)

Table S14 Cox hazard ratio for Male in Period 3

	Univariate			Multivariate		
Term	Hazard ratio	95% C.I.	p-value	Hazard ratio	95% C.I.	p-value
COVID-19 infection	0.44	0.15-1.29	0.143	0.50	0.16-1.55	0.23
Age				1.05	0.99-1.11	0.081
Degree of Care						
Moderate				3.28	0.37-29.20	0.29
High				4.67	0.52-41.63	0.17
CCI				1.04	0.85-1.28	0.71

Number of Vaccinations	0.71	0.47-1.09	0.12

Table S15 Characteristics of inter-period comparison for Male

Characteristic	Period 1 (n=234) [1]	Period 2 (n=275) [1]	Period 3 (n=286) [1]
Age	86(7)	87(7)	87(6)
Person-year	0.90 (0.71)	0.68 (0.65)	0.60 (0.53)
Number of Deaths	35 (41%)	34 (37%)	36 (35%)
Degree of care			
Low	13 (15%)	13 (14%)	15 (15%)
Moderate	32 (38%)	45 (49%)	42 (41%)
High	40 (47%)	34 (37%)	46 (45%)
CCI	2.16 (1.48)	1.79 (1.31)	1.79 (1.30)

Period 1:2018-2019, Period 2:2020-2021, Period 3:2022-2023

[1] (SD); n (%)

[2] One-way analysis of means (not assuming equal variances); Fisher's exact test

Table S16 Cox Hazard test for inter-period comparison for Male

	Univariate			Multivariate		
Term	Hazard ratio	95% C.I.	p-value	Hazard ratio	95% C.I.	p-value
Period1 vs Period2	1.18	0.74-1.86	0.98	1.11	0.70-1.75	>0.99
Period1 vs Period3	1.27	0.79-2.04	0.96	1.18	0.74-1.89	>0.99
Period2- vs Period3	1.08	0.65-1.80	0.98	1.06	0.65-1.73	>0.99
Age				1.03	1.01-1.06	0.015
Degree of Care						
Moderate				1.44	0.75-2.81	0.27
High				2.12	1.11-4.06	0.023
CCI				0.99	0.87-1.11	0.82

Table S17 Female

**Characteristic**	uninfected (n=311)[1]	infected (n=212)[1]	p-value[2]
Number of deaths	70 (23%)	34 (16%)	0.075
Age	89.0(7.0)	88.0(6.0)	0.13
Degree of care			0.50
Low	24 (8%)	23 (11%)	
Moderate	157 (37%)	101 (48%)	
High	130 (42%)	88 (42%)	
CCI	1.16 (0.88)	1.24(0.91)	0.30
Number of vaccinations			0.001
0	103 (33%)	43 (20%)	
1	5 (1.6%)	1 (0.5%)	
2	202 (65%)	105 (78%)	
3	1 (0.3%)	3 (1.4%)	

[1] n (%); Mean (SD)

[2] Fisher's exact test; One-way analysis of means (not assuming equal variances)

Table S18 Cox hazard ratio for Female in Period 3

	Univariate			Multivariate		
Term	Hazard ratio	95% C.I.	p-value	Hazard ratio	95% C.I.	p-value
COVID-19 infection	1.42	0.92-2.21	0.12	1.62	1.03-2.54	0.035
Age				1.08	1.03-1.12	<0.001
Degree of Care						
Moderate				1.63	0.59-4.49	0.35
High				3.52	1.30-9.50	0.013
CCI				1.14	0.90-1.45	0.29
Number of Vaccinations				0.89	0.69-1.16	0.12

CCI stratified by survival

Table S19 Comparison of CCI in period 1-3

Period	Event	CCI
Period1	survived (n=380)	1.57 (1.16)[1]
	deceased (n=149)	1.66 (1.38)[1]
Period 2	survived (n=417)	1.33 (1.01)[1]
	deceased (n=172)	1.46 (1.12)[1]
Period 3	survived (n=491)	1.30 (0.99)[1]
	deceased (n=139)	1.58 (1.20)[1]

[1] mean (SD)

Table S20 Cox hazard test for CCI of period1

	Univariate			Multivariate		
Term	Hazard ratio	95% C.I.	p-value	Hazard ratio	95% C.I.	p-value
CCI	1.10	0.96-1.25	0.71	1.05	0.93-1.35	0.911
Age				1.06	1.03-1.09	<0.001
Sex				2.07	1.57-3.49	<0.001

Table S21 Cox hazard test for CCI of period2

	Univariate			Multivariate		
Term	Hazard ratio	95% C.I.	p-value	Hazard ratio	95% C.I.	p-value
CCI	1.03	0.89-1.19	0.71	0.99	0.85-1.15	0.44
Age				1.06	1.03-1.09	<0.001
Sex				2.34	1.57-3.49	<0.001

Table S22 Cox hazard test for CCI of period 3

	Univariate			Multivariate		
Term	Hazard ratio	95% C.I.	p-value	Hazard ratio	95% C.I.	p-value
CCI	1.26	1.08-1.45	0.003	1.16	1.00-1.34	0.045
Age				1.06	1.03-1.10	<0.001
Sex				3.22	2.14-4.86	<0.001

Comparison of survival rate

Table S23 Survival rate between period1-3 by eliminating period after COVID-19 infection in Period 3

Characteristic	Period 1 (n=529) [1]	Period 2 (n=589) [1]	Period 3 (n=630) [1]	p value[2]
Age	88 (7)	89(6)	89 (6)	0.50
Sex	85 (16%)	92 (16%)	103 (16%)	>.99
Person-year	1.07 (0.73)	1.01 (0.74)	0.79 (0.63)	<0.001
Number of Deaths	149 (28%)	172 (29%)	103 (16%)	<0.001
Degree of care				0.40
Low	63 (12%)	78 (13%)	63 (10%)	
Moderate	236 (43%)	270 (46%)	302 (48%)	
High	230 (43%)	241 (41%)	265 (42%)	
CCI	1.60 (1.19)	1.37 (1.04)	1.36 (1.05)	<0.001

Period 1:2018-2019, Period 2:2020-2021, Period 3:2022-2023

[1] Mean (SD); n (%)

[2] One-way analyses of means (not assuming equal variances); Fisher's exact test with Holm test

Table S24 Cox Hazard ratio test for inter-period comparison between period1-3

	Univariate			Multivariate		
Term	Hazard ratio	95% C.I.	p-value	Hazard ratio	95% C.I.	p-value
Period 1 vs Period 2	1.09	0.88-1.36	0.43	1.09	0.88-1.36	0.43
Period 1 vs Period 3	0.77	0.59-0.99	0.087	0.77	0.59-0.99	0.084
Period 2 vs Period 3	0.70	0.54-0.90	0.016	0.82	0.54-0.90	0.015
Age				1.06	1.04-1.07	<0.001
Sex				2.51	1.96-3.22	<0.001
Degree of Care						
Moderate				1.59	1.03-2.47	0.038
High				3.09	2.02-4.73	<0.001
CCI				1.07	0.99-1.15	0.090
